# Supplementary material for: Resolving the full spectrum of human genome variation using Linked-Reads
Source: Genome Res. 2019 Apr;29(4):635–45. doi: 10.1101/gr.234443.118 (PMC6442396; doi:10.1101/gr.234443.118)
Supplement: Supplemental Material [file supp_gr.234443.118_Supplemental_longranger-2.2.zip › longranger-2.2.tar.bz2!/lr2.2-public/lib/stan/cmdstan/stan/doc/api/html/cmd__line_8hpp.html]

Stan: src/stan/io/cmd\_line.hpp File Reference


|  |  |
| --- | --- |
|  | Stan  2.5.0  probability, sampling & optimization |


- Main Page
- Related Pages
- Namespaces
- Classes
- Files

- File List
- File Members

All Classes Namespaces Files Functions Variables Typedefs Enumerations Enumerator Friends Macros Pages

- src
- stan
- io

Classes |
Namespaces |
Functions

cmd\_line.hpp File Reference

`#include <map>`  
`#include <ostream>`  
`#include <set>`  
`#include <string>`  
`#include <sstream>`  
`#include <vector>`

Go to the source code of this file.

|  |  |
| --- | --- |
| Classes | |
| class | stan::io::cmd\_line |
|  | Parses and stores command-line arguments. More... |
|  | |

|  |  |
| --- | --- |
| Namespaces | |
|  | stan |
|  | Probability, optimization and sampling library. |
|  | |
|  | stan::io |
|  | Input/output utilities. |
|  | |

|  |  |
| --- | --- |
| Functions | |
| void | stan::io::pad\_help\_option (std::ostream \*o, const std::string &option="", unsigned int width=20) |
|  | Print help option with padding. More... |
|  | |
| void | stan::io::print\_help\_helper (std::ostream \*o, const std::string &key\_val, const std::string &msg, const std::string &note="") |
|  | Prints single print option to output ptr if non-null. More... |
|  | |
| void | stan::io::print\_help\_option (std::ostream \*o, const std::string &key, const std::string &value\_type, const std::string &msg, const std::string &note="") |
|  | Prints single print option to output ptr if non-null. More... |
|  | |
| template<> | |
| bool | stan::io::cmd\_line::bare< std::string > (size\_t n, std::string &x) const |
|  | |
| template<> | |
| bool | stan::io::cmd\_line::val< std::string > (const std::string &key, std::string &x) const |
|  | |


---

[ Stan Home Page ]

*© 2011–2014,
Stan Development Team.*
